# Marmoset prosociality is intentional

**DOI:** 10.1007/s10071-020-01363-6

**Published:** 2020-02-27

**Authors:** Judith M. Burkart, Carel P. van Schaik

**Affiliations:** Department of Anthropology, University of Zurich, Winterthurerstrasse 190, 8057 Zürich, Switzerland

**Keywords:** Proactive prosociality, Common marmosets (*callithrix jacchus*), Intentionality, Goal-directedness, Audience effects, Flexibility

## Abstract

Marmoset monkeys show high levels of proactive prosociality, a trait shared with humans, presumably because both species rely on allomaternal care. However, it is not clear whether the proximate regulation of this convergent trait is also similar, in particular with regard to intentionality, which is a defining characteristic of prosocial behavior in the human literature. The aim of this paper was to investigate whether marmoset monkeys’ prosociality fulfils the criteria of intentionality developed in primate communication research. The results show that marmoset prosocial behavior (i) has some degree of flexibility, since individuals can use multiple means to reach their goal and adjust them to specific conditions, (ii) depends on the presence of an audience, i.e. potential recipients (social use), and (iii) is goal-directed, because (a) it continues exactly until the putative goal is reached, and (b) individuals check back and look at/for their partner when their prosocial actions do not achieve the putative goal (i.e. if their actions don’t lead to the expected outcome, this elicits distinct reactions in the actor). These results suggest that marmoset prosociality is under some degree of voluntary, intentional control. They are in line with other findings that marmosets perceive each other as intentional agents, and only learn socially from actions that are perceived as intentional. The most parsimonious conclusion is, therefore, that prosocial behavior is fundamentally under voluntary control in marmosets, just as it is in humans, even though our more sophisticated cognitive abilities allow for a far more complex integration of prosociality into a broader variety of contexts and of behavioral goals.

## Introduction

**Fig. 1:**
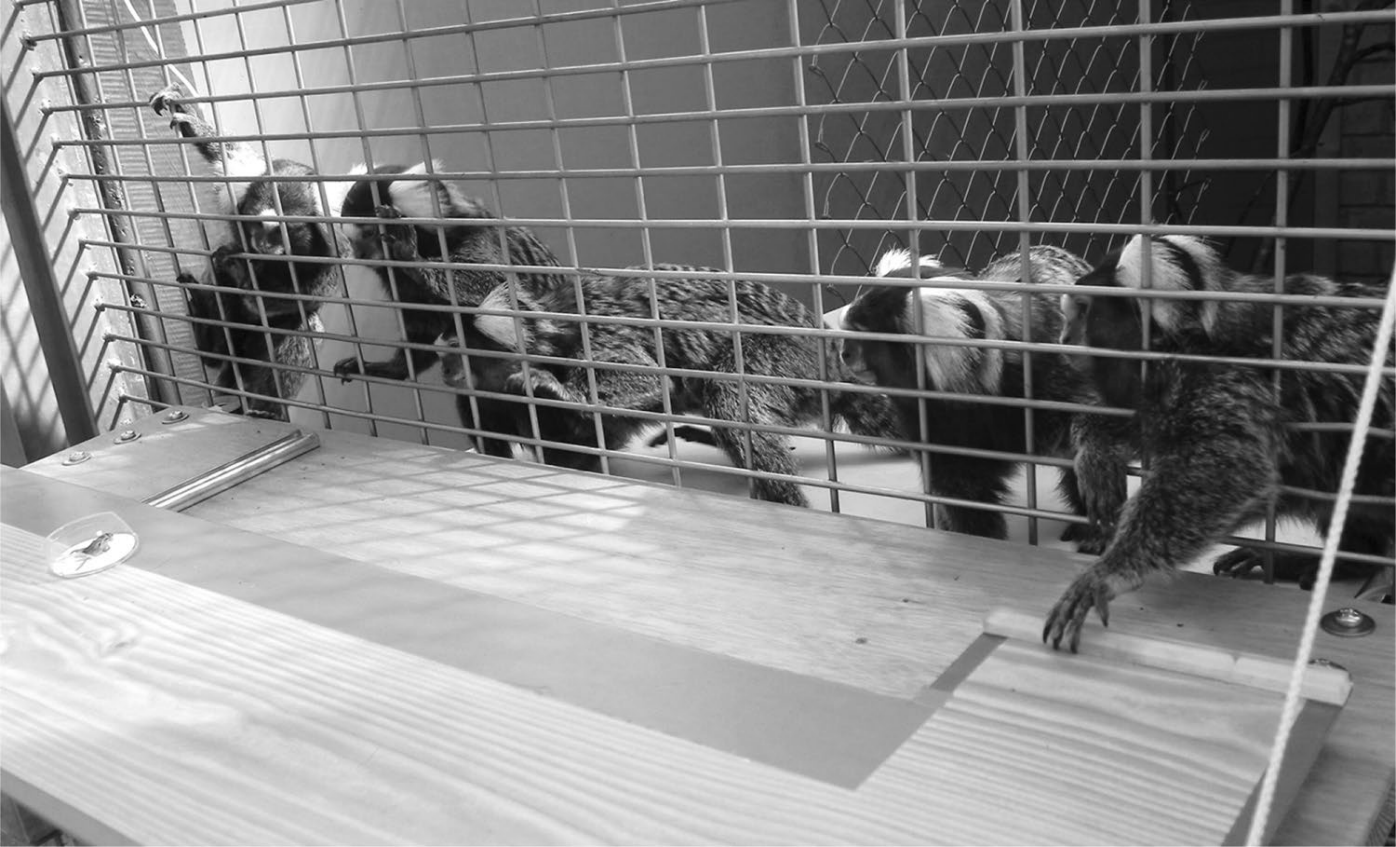
Experimental setup of the group service experiments. See also video S1. A board with food on top of it (cricket in the white bowl on the left-hand side) is attached to the home enclosure and can be pulled closer to the wire mesh, which makes the food available for the rest of the group. The animal on the right side pulls the board within reach, allowing other group members to retrieve the food from the board. Coordinated pulling is necessary, however, because in contrast to the dyadic game, the board rolls back out of reach automatically if it is released

**Fig. 2:**
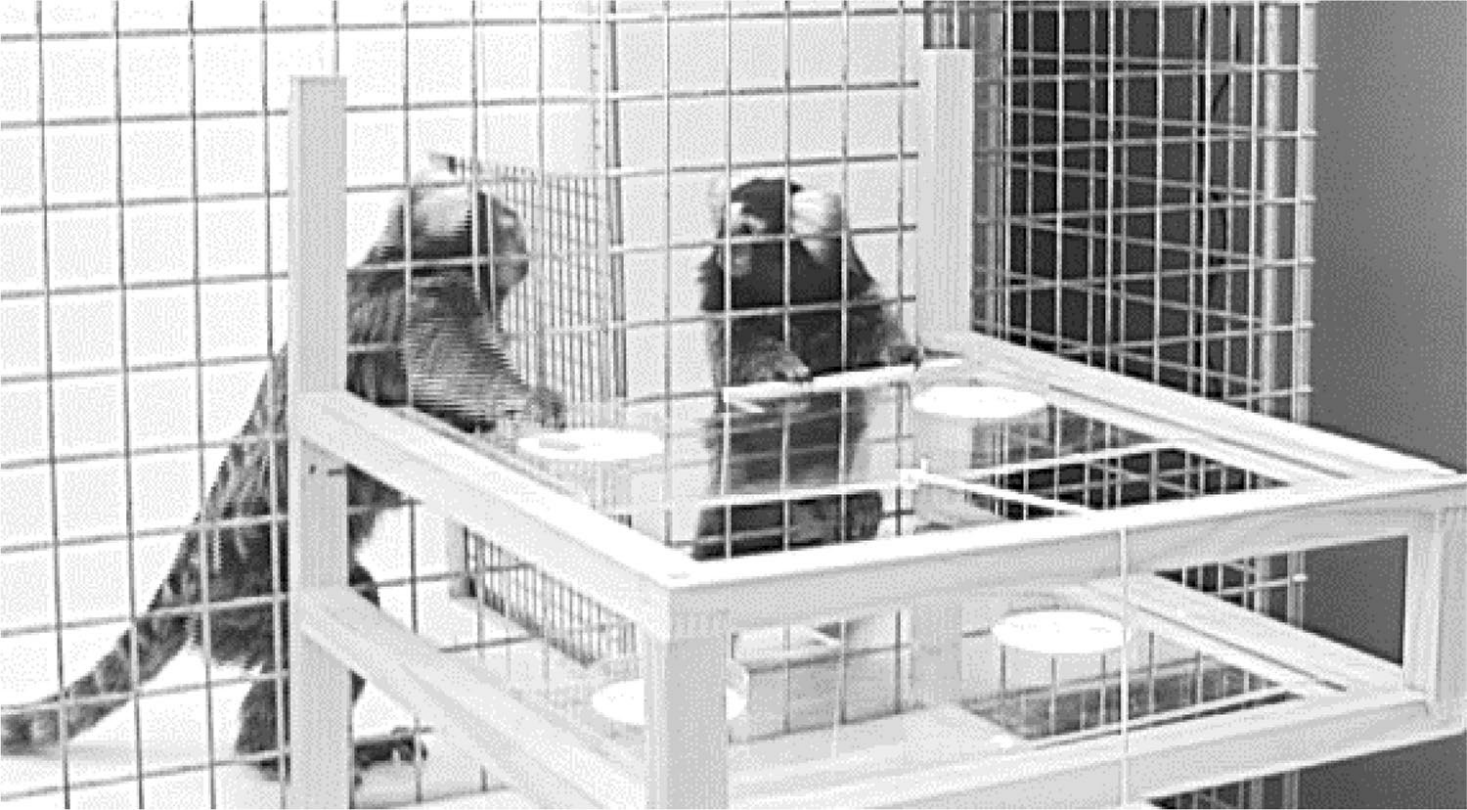
Experimental setup of the dyadic games. See also video S2. The donor individual in the right-hand compartment can pull the upper or lower tray within reach of the recipient individual in the left-hand compartment (who is grasping for the food reward in the upper left food bowl, on the tray that is being pulled by the donor). The recipient cannot pull the trays because she has no access to the handle. An individual’s prosociality refers to the pulling frequency when a partner is present minus the pulling frequency in the control condition when no partner is present. Note that once a tray is pulled, it stays close to the wire mesh, so the recipient has access to the food bowl

Common marmosets (*Callithrix jacchus*) are cooperatively breeding callitrichid New World monkeys. Like other callitrichids, they live in cohesive family groups who cooperate in a variety of contexts, including group defense, food acquisition and caring for immatures (Digby et al. 2007; Erb and Porter 2017; Garber 1997; Yamamoto et al. 2014). Caretaking includes both infant carrying and sharing of food, which is frequent and performed by all group members, who in captivity may share up to 80% of all food items they obtain in food sharing tests (Finkenwirth et al. 2016; Guerreiro Martins et al. 2019). Food sharing can be reactive, where the adult tolerates the immature to take her food, but is also often proactively offered. In proactive food sharing or offering, the initiative for the food transfer is taken by the food possessor, who holds the food in its hand or mouth, calls the immatures by emitting a food call, and waits for them to come and get the food (Brown et al. 2004). Together, these cooperative behaviors suggested that they may be motivated by other-regarding preferences (Fehr and Fischbacher 2003) or proactive prosociality, i.e. a concern not only for one’s own, but also for others’ welfare (Hrdy 2005; Silk et al. 2005; Snowdon and Cronin 2007). Accordingly, experimental evidence has shown that adult marmosets indeed show proactive prosociality, in dyadic provisioning games (Burkart et al. 2007) and in group service experiments (Burkart et al. 2014; see Figs. 1 and 2). To investigate the evolutionary origin of proactive prosociality, the latter experiments were also performed with a variety of other primate species, including humans, and the results revealed that the extent of a species’ prosociality is correlated with the amount of help mothers receive when rearing the offspring. Humans thus cluster together with callitrichid monkeys, with both showing high levels of proactive prosociality in the group service experiment and high levels of allomaternal care, whereas chimpanzees, our closest relatives, show far lower levels in both measures.

The high levels of proactive prosociality in callitrichid monkeys and humans is thus best understood as a convergent adaptation to allomaternal care (but see also Thornton and McAuliffe 2015, and Burkart and van Schaik 2016), which raises the question of how similar prosociality in callitrichids and humans really is, and whether it is regulated by the same proximate mechanisms. At the level of hormonal regulation, recent efforts indeed suggest several parallels with regard to the neuro-hormone oxytocin (Freeman and Bales 2018; Marsh 2019; Mustoe et al. 2015; Saito and Nakamura 2011). For instance, oxytocin is correlated with proactive food sharing with immatures (Finkenwirth et al. 2016), and also with the presence of strong bonds and proactive prosociality among adult marmosets (Finkenwirth and Burkart 2017).

Less is known regarding the underlying cognitive mechanisms, in particular whether prosocial behaviors in marmosets are under voluntary intentional control, which is among the critical defining criteria for prosociality in the human literature (Batson et al. 2008; Eisenberg et al. 2006, 2016; Eisenberg and Miller 1987; Hawley 2014). For instance, in psychology, a behavior will qualify as prosocial only if it was *intended* to be beneficial for someone else, even if the goal is not attained. In animal studies, however, the question of intentionality in prosocial behavior has never been asked explicitly. Our goal is to fill this gap, and to do so we can build on the considerable body of research in animal communication, which has successfully developed criteria to identify intentionality in communicative signals in nonhuman animals (Hobaiter and Byrne 2014; Liebal et al. 2013; Schel et al. 2013; Townsend et al. 2017; Ben Mocha and Pika 2019). We, therefore, adopt this approach here to investigate to what extent marmoset proactive prosociality, as shown under naturalistic conditions and in several experimental contexts, is under voluntary control and pursues social goals, to evaluate how comparable the proximate, mechanistic regulation of this convergent trait is in humans and marmosets.

In nonhuman primates, the intentionality question has traditionally been addressed in the context of communication, in particular gestural communication (Hobaiter and Byrne 2014; Hopkins et al. 2007; Leavens et al. 2005; Liebal et al. 2013; Roberts et al. 2013; Schel et al. 2013; Tomasello et al. 1994) but also in the context of understanding others’ actions (Burkart et al. 2012; Call et al. 2004; Phillips et al. 2009; Rochat et al. 2008; Wood et al. 2007). The approach to identify intentionality in communicative signals has been to formulate criteria that must be fulfilled for the signal to be counted as intentional (e.g. summarized in Liebal et al. 2013). These criteria are called markers of intentionality, and include for instance flexibility, social use and audience effects, sensitivity to the attentional state of the recipient, the use of attention getters, audience checking, persistence, and elaboration. For instance, Cartmill and Byrne (2010; see also Hobaiter and Byrne 2014) identified a communicative gesture as intentional if it fulfilled at least one of the following criteria: flexible rather than automatic production, social use (i.e. the signaler directs the behavior toward another and shows signs of being aware of the potential recipients and their state of attention: so-called audience effects) and goal-directedness (i.e. the signaler expects a reaction to the gesture and persists in signaling, or engages in response waiting, by pausing at the end of the communication and maintaining some visual contact).

There are different ways to summarize markers of intentionality in broader criteria. For instance, Cartmill and Byrne (2010) use the broad criteria flexibility, audience effects, and directedness toward social goals, each of which can be satisfied with various markers of intentionality. Townsend et al. (2017) use the three criteria goal-directedness, recipient-directedness, and that the result should be a change in the behavior of the recipient which is consistent with the goal. Again each of these three criteria can be validated with different markers of intentionality, which largely overlap with the markers referred to by Cartmill and Byrne 2010. There are also different claims with regard to whether a communicative act has to show all possible markers of intentionality, or at least one for each criterion, or whether a single marker is enough (summarized in Townsend et al. 2017). Although a definitive decision on these issues may not yet be possible, here we adopt the following underlying rationale. Because every single marker of intentionality is vulnerable to alternative, low-level explanations, the more markers, i.e. the more convergent evidence is available which is consistent with an intentional description, the more confident one can be that such a description is indeed the most accurate one (see also Townsend et al. 2017). Importantly, many of the criteria developed for intentionality in communication (e.g. Dennett 1983) can readily be applied to non-communicative behaviors as well (e.g. Canteloup et al 2017).

In the context of marmoset food sharing, in naturalistic and experimental contexts, the intentionality question has rarely been addressed explicitly. Nevertheless, when looking for markers of intentionality as developed for animal communication, it quickly becomes evident that many of the patterns reported in the literature correspond to such markers of intentionality. In this paper, we, therefore, summarize these findings, highlighting what markers of intentionality are already available from published studies (the regular entries in Table 1), and add evidence for additional markers of intentionality, through re-analysis of videos of previous experiments and additional empirical data (the ***bold italic*** entries in Table 1). In doing so, we lay out how much convergent evidence for intentionality is available for marmoset food sharing.

**Table 1:** Criteria for intentionality in marmoset prosociality

Criteria and Markers	Evidence
FLEXIBILITY (INSTRUMENTAL GOALS)	
Behavioral adjustment to specific conditions	Naturalistic behavior in wild and captivity: more proactive food sharing when food is novel or difficult to obtain for immatures^1–7^
Means-end dissociation	
i.e. to be able to use multiple means to achieve a goal	Dyadic game and group service: proactive prosociality not only expressed in proactive food sharing under naturalistic conditions, but also in experimental contexts requiring novel actions^8,9^
AUDIENCE EFFECTS	
Audience effects I i.e. to only show the behavior when an audience (recipient) or a specific audience (composition) is present	Naturalistic context: more food calls when others are out of sight but within earshot, to inform them about the presence of food^10,11^ (recipient) or when immatures are in the group (composition)^12–14^
	Experimental contexts: more pulling when partner(s) is present than when absent in dyadic games^8^*** and group service (this study)***
Audience effects II	
i.e. whether the presence of additional potential helpers influences helping	Experimental context: more proactive sharing with immatures when it alone is responsible for *them*^*15*^
GOAL-DIRECTEDNESS (SOCIAL GOALS)	
***Persistence*** ***i.e. behavior continues until putative goal (response) is reached***	***Experimental group service context: the behavior “holding the tray” continues exactly until the goal (taking the food by recipient) is reached (this study)***
***Audience checking*** ***i.e. show a distinct reaction when the behavior does not result in the putative goal***	***Experimental Dyadic games: prosocial individuals check back and look at/for their partner when they have pulled food to within reach for them but the partner does not retrieve the food (this study)***

Entries refer to published evidence showing how these criteria are met by marmoset prosociality in naturalistic and experimental contexts; entries in ***bold italics*** refer to evidence presented in this study

^1^Guerreiro Martins and Burkart (2013)

^2^Humle and Snowdon (2008)

^3^Moura et al. (2010)

^4^Price and Feistner (1993)

^5^Rapaport (2011)

^6^Yamamoto et al. (2014)

^7^Rapaport (1999)

^8^Burkart et al. (2007)

^9^Burkart et al. (2014)

^10^Vitale et al. (2003)

^11^Caine et al. (1995)

^12^Feistner and Price (1991)

^13^Joyce and Snowdon (2003)

^14^Rapaport (2011)

^15^Brügger et al. (2018)

There are important differences between mere intentional behaviour and intentional communication (Bard 1992). Marmoset food sharing, we argue, is something in between: on one hand, it consists of an instrumental goal such as making sure the food ends up in a specific location (e.g. in Figs. 1 and 2). On the other hand, it should also fulfil certain social goals—e.g. to make sure food only ends up in this specific location when a potential recipient is indeed present, provision of more food, when no one else is around, to help a potential recipient, to persist in the behavior until the food has been taken, or to expect that the food will indeed be taken by a social partner and be surprised if it doesn’t happen so. Several of the criteria from the communication literature can thus be applied to marmoset food sharing, in naturalistic and experimental contexts (whereas others obviously cannot, such as having a communicative intent or specific referent, see also Townsend et al. 2017 for details). In Table 1, we present markers of intentionality that are applicable to marmoset food sharing according to the classification of Cartmill and Byrne (2010), and summarize predictions that would support flexibility (instrumental goals), different kinds of audience effects, and directedness toward social goals.

Accordingly, we summarize criteria that should be met if the behavior is intentional in the sense of reaching the instrumental goal under the first header (flexibility), and of reaching a social goal under the second and third header. Note that the classification of Cartmill and Byrne (2010) we are using here largely overlaps with the one from Townsend et al. (2017). The latter, however, also stresses that in the recipient, the communicative act should lead to a behavioral change that is consistent with the supposed goal. In the context of food sharing, this goal would be that a recipient who is offered the food indeed takes the food. This is rather trivial, which is why we don’t further elaborate on this here and stick to the criteria from Cartmill and Byrne (2010). More telling in the food sharing or experimental prosociality contexts are situations where offered food is *not* taken. If food donors respond to this situation with surprise, this would be a particularly strong evidence for pursuing a social goal because it suggests that donors indeed expect the recipient to take the food that is offered to them (see below).

The additional tests (the ***bold italic*** entries in Table 1) were developed in the context of the experimental marmoset prosociality paradigms, i.e. dyadic prosociality games and group service experiments, by running additional experimental conditions (test 1) or by re-analyzing available data with a focus on these questions (tests 2 and 3). Each of these paradigms offers the opportunity to address a specific set of intentionality markers, as outlined in Table 1.

A first criterion for intentional instrumental behavior is that the action under investigation should be used flexibly rather than produced automatically. Flexibility includes that the behavior is adjusted to specific conditions, e.g. that proactive food sharing is not simply triggered automatically by the presence of immatures of a specific age. Variation in the amount of food sharing under naturalistic conditions is consistent with this aspect of flexibility*.* For instance, adults are more likely to share a food if it is novel to the immature rather than familiar (Rapaport 1999), or if the food has to be retrieved from a puzzle feeder that only the adult is able to solve, and if the adult witnesses that the immature is unable to do so, compared to an identical food item that is simply handed over to the adult (Guerreiro Martins and Burkart 2013; see also Humle and Snowdon 2008; Moura et al. 2010; Price and Feistner 1993; Rapaport 2011 for similar findings in tamarins in the wild and in captivity). In other words, adults appear to take the immature’s need and skills in obtaining food into account when deciding whether to share or not, and can do so independently of the immature’s age.

Another aspect of flexibility is that the same instrumental goal can be achieved by different means (means-end dissociation, i.e. intentional behavior sensu Piaget 1954 and Bard 1992). Thus, proactive food sharing should not only occur in the form of the behavioral pattern described above, which arguably could be an automatically triggered response to finding food when immatures are present in the group. Rather, it should likewise occur in evolutionarily novel contexts, such as experimental prosociality tasks which require fundamentally different and highly artificial behavioral responses, e.g. pulling a tray within reach of a partner. Experimental evidence for proactive prosociality in common marmosets (Burkart et al. 2007; Burkart et al. 2014) satisfies this aspect of flexibility, and, therefore, suggests that donors represent the goal of provisioning rather than engage in stimulus-elicited responses.

All subsequent criteria are concerned with social goals. Thus, a second criterion for intentionality that can be adopted for proactive prosociality is that the behavior should be sensitive to the *presence of an audience*, also referred to as social use. Under naturalistic conditions, audience effects on food calls among adults have been reported for common marmosets (Vitale et al. 2003) and red-bellied tamarins (Caine et al. 1995). In both species, individuals emitted more food-offering calls when no partner was immediately present, but still within earshot. The food calls are thus specifically given for those who can hear them, but cannot see the food themselves, which is fully consistent with an intentional description because for those who are close by and can see the potential caller and the food immediately, additional information is not necessary to inform them about the presence of the food. The function of these food-offering calls is to attract others to the food source, since visually absent individuals are still likely to be within hearing distance under naturalistic conditions. Note that this pattern contrasts with other species in which food calls have different functions, as for instance capuchin monkeys, who have been reported to call more when an immediate audience was visually present, which suggests that their food calls function more to defend, rather than offer food (Gros-Louis 2004; Pollick et al. 2005), or alternatively to avoid aggression upon detection (punishment: Raihani et al. 2010).

The *presence of an audience* also matters in experimental dyadic prosociality games (see below for details). In fact, the difference of pulling the baited tray when a partner is present compared to a non-social control condition is the key criterion for showing proactive prosociality in such games (Cronin 2012; Marshall-Pescini et al. 2016). In the group service experiment (see below for details), a different approach was used to assess proactive prosociality in their social group, i.e. without separating individuals (Burkart et al. 2014, see also SI in Burkart and van Schaik 2016). The group service experiment can, however, be modified to more directly assess audience effects. This was the first goal of our study (Test 1). We collected data in a condition in which an audience in the form of potential recipients was present (i.e. a group of common marmosets had access to the entire home enclosure) or absent (i.e. the group was prevented from accessing the part of the home enclosure where the food would be provisioned). We, therefore, predicted that the group would pull more in the full-access condition compared to the condition where the group members had no access to the food-provisioning site.

For marmoset food calls, the *composition of the audience* matters too. Food-offering calls are more frequently given when immatures are present in the social group (Feistner and Price 1991; Joyce and Snowdon 2003; Rapaport 2011). Likewise, in dyadic games, the identity of the partner matters: closely bonded partners, who show synchronized oxytocin fluctuations, are more likely to behave prosocially (Finkenwirth and Burkart 2017).

*Audience effects* on food sharing can also be investigated *with regard to additional, not directly involved bystanders*. Brügger, Kappeler-Schmaltzried and Burkart (2018) tested whether focal marmosets were more likely to share food with immatures when other group members were also present compared to when they were alone with the immatures. When observed with by bystanders, focals may share more to engage in reputation management (e.g. pay-to-stay in the case of helpers), or because they are subjected to subtle forms of coercion. Instead, all marmosets, i.e. helpers and breeders of both sexes, showed a strong audience effect in the opposite direction: they shared more, rather than less, when they were alone with the immatures. This reaction is consistent with the so-called diffusion of responsibility effect well known in humans (Bierhoff and Rohmann 2016), and corroborates the strong concern of all group members for the immatures, in particular when they are solely responsible for them.

A third criterion to assess intentionality that can be adopted for prosocial behaviour is that the behavior should be goal-directed with regard to social goals, which in this case is the benefit of the recipient. This leads to two predictions. First, if the behavior is goal-directed, it should be persistent and last exactly until the goal is reached. This prediction can only be tested in the group service context, where an individual has to pull the apparatus and *hold it until* a second individual retrieves the food, because otherwise, the apparatus automatically rolls back and the food is again out of reach. Our second goal, therefore, was to analyze whether pulling in group service was persistent and ended exactly when the goal of provisioning was achieved (Test 2). We, therefore, reanalyzed the data from Burkart et al. (2015) to assess whether pulling would indeed stop exactly when the recipients would have taken the food from the board.

A second prediction of goal-directedness was that subjects should show a distinct reaction if the behavior occasionally would not result in the putative goal. The dyadic prosocial game offers the opportunity to test this prediction because here, the tray would not roll back automatically once pulled. In this game, it could, therefore, happen that a donor would pull the tray for the recipient, but for some reason, the recipient would not retrieve the food. We predicted that in such cases, a prosocial donor would check back at the recipient individual by looking at it or for it (i.e. audience checking), and that the strength of this reaction to an unexpected outcome would be related to an individual’s level of prosociality. This prediction was investigated in test 3, based on a re-analysis of the looking behavior in Burkart et al. (2007).

## Methods

To test the predictions outlined above, we conducted additional experimental conditions in the context of the group service paradigm (test 1) and performed detailed behavioral analyses of already available data from two paradigms that have been used to quantify proactive prosociality in common marmosets: group service (test 2) and dyadic prosocial games (test 3). We start by giving an overview for these paradigms (the full methodological details for these tasks are available in Burkart et al. (2007), and Burkart and van Schaik (2013) and Burkart et al. (2014), respectively). We then proceed with the detailed methods for Tests 1–3.

### Group service paradigm

In the group service experiments, the subjects were tested while in their social group in their home enclosure. The apparatus consisted of a wooden board in front of the wire mesh of the home enclosure (Fig. 1). A food bowl was attached on one end of the board, whereas a handle was available at the far end. By pulling the handle, an animal can pull the food bowl towards the enclosure. However, because the board rolls back to its far away starting position as soon as the handle is released, the pulling individual cannot itself obtain the food because the food bowl is too far away from the handle. To successfully provide food to group members, an individual thus has to pull the board and hold it until another individual has taken the food. The subjects were first trained to understand how the apparatus worked and once all subjects had passed the corresponding criteria, the test phase started. During 10 days, they were presented with 85 trials per day. Every other day was an experimental day, and in regular trials (blocks of five trials), food was in the bowl at the end of the board, whereas in the remaining trials in between, food was in a bowl close to the handle. On control days, in regular trials the food bowls were empty but made salient by tapping on them, whereas in the remaining, the so-called motivation trials the food was again close to the handle.

Proactive prosociality was quantified as the percentage of food made available in the test trials of the experimental sessions four and five (see Burkart and van Schaik 2013 and Burkart et al. 2014 for all experimental details and results). For the scope of species comparison, this measure is calculated as a group measure (see Burkart et al. 2014), but individual donor measures (i.e. the percentage of food made available per individual) from the group service paradigm are positively correlated with individual donor measures from dyadic games (averaged across dyad partners; see Burkart and van Schaik 2013).

### Dyadic prosociality games

In the dyadic games, dyads of individuals are tested in an experimental enclosure, in two compartments separated by an opaque divider (Fig. 2). A part of the divider, i.e. the window, is made of wire mesh, allowing the animals to see each other. An apparatus in front of the cage is made of two drawers, but the handle to pull the drawer within reach is only available on one side (to the potential donor, i.e. the individual on the right-hand side in Fig. 2). When a piece of food is placed in front of the food bowl of the potential recipient (i.e. the individual on the left-hand side), the donor can pull the tray for the recipient who then can take the food. In the control condition, no partner is present. We conducted 18 trials per dyad. Importantly*, proactive prosociality* is calculated as a *difference score,* i.e. the difference of pulling the baited tray on the recipient side in test sessions minus pulls in the control sessions. Prior to the experiment, all individuals had to reach several criteria to make sure they understood the consequences of their pulling. We reanalyzed the behavior of 29 dyads in this game, composed of 10 individuals (the Kalium group from Burkart et al. 2007), who as a group pulled the tray significantly more in test sessions compared to control sessions and for whom alternative explanations such as social facilitation or contagious reaching had been excluded empirically.

### Method test 1: audience effects in the group service paradigm

**Fig. 3:**
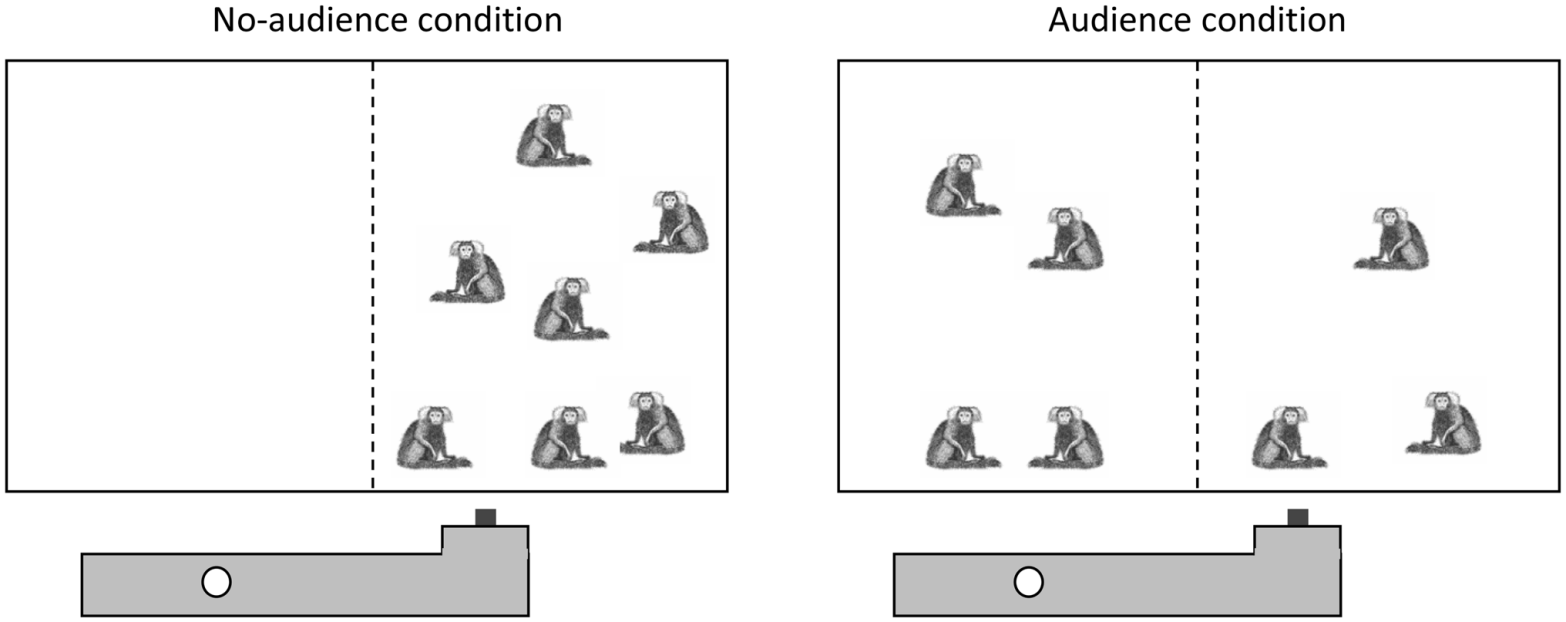
Setting to assess audience effects in the group service task. The home cage is separated in two compartments. If the subjects are sensitive to whether a recipient of a potential provisioning act is present or not, they should be more likely to pull the board in the audience condition compared to the no-audience condition

We tested common marmosets in an additional condition of the group service paradigm (a group composed of a breeding pair, five adult male helpers and one adult female helper (from Burkart and van Schaik 2013). To assess audience effects, i.e. whether marmosets would only pull the tray when a partner was present, the home cage was separated in two parts (Fig. 3). In the no-audience condition, the whole group was on one side and the apparatus would deliver food to the empty compartment when pulled, whereas in the audience condition, half of the group was in the second compartment and could be provisioned by group members.

If the animals were sensitive to the presence of potential recipients of their provisioning actions (i.e. the audience), pulling should be far more frequent (and significantly so) during the audience-present condition. These additional experiments were run directly after the group service experiment.

Each condition was tested during 25 trials, and the no-audience condition was presented first. Each trial lasted for 60 s, or less in the audience condition when the food had been provisioned earlier. For each trial, every pull made by an individual was recorded. In the audience-present condition, it was also recorded whether a pull led to a food transfer or not. Since the behavior of the individuals is not independent from each other, the pulling frequency was compared at the group level. Note that this biases the test against the prediction since in the audience-present condition, fewer individuals were present in the donor compartment and thus fewer could potentially pull. In addition, we compared the number of provisioned food items in the audience-present condition with the number of items provisioned during the last regular test session of the same group reported by Burkart and van Schaik (2013).

### Method test 2: persistence in the group service paradigm

The goal of this re-analysis was to quantify whether marmosets would persist to hold the board tightly close to the mesh exactly until a group member would have retrieved the reward. This criterion could only be assessed in the group service context because only here the board had to be held tightly to prevent it from sliding back again. We analyzed the timing of the food transfer relative to the pull duration in phase IV in the Jojoba group (one breeding pair, two adult female helpers and two adult male helpers) in the videos from Burkart et al. (2014). Pull durations are variable, but if a pull is performed with the goal of providing the food to group members, one would expect that it ends right after the recipient has taken the food. Alternatively, if the subjects pulled the tray for any reason other than providing the food, the transfers should be distributed randomly over the period when the tray was pulled. We measured the full duration of each pull that led to a transfer, and the latency from the beginning of the pull to when the transfer occurred. We then calculated the number of transfers that occurred during the first 20% of the duration of a pull, during the second 20%, as well as the third, fourth and fifth 20% and compared this distribution to the expectation of a random distribution. A second coder coded 20% of all trials, and the reliability of the duration of a pull was Rho = 0.841, *n* = 57, *p* < 0.001, and the transfer latency relative to the start of a pull was Rho = 0.694, *n* = 57, *p* < 0.001.

### Method test 3: are marmosets surprised if the food they provide to a partner is not taken? Audience checking

**Fig. 4:**
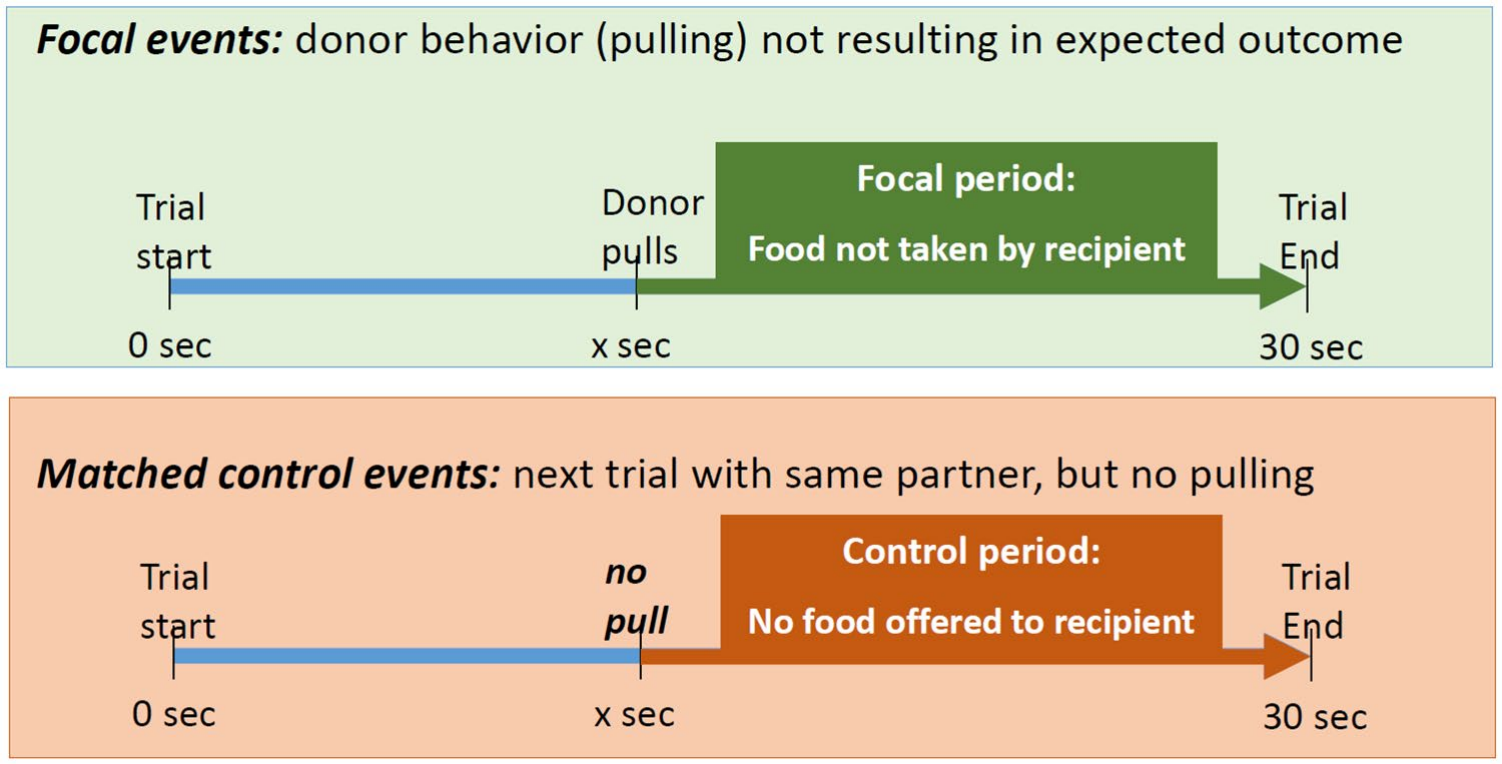
Illustration of the focal and matched control periods in test 3. In both the focal and the control periods, we quantified how often the donor would look at, and look for, the partner. The strength of the reaction of donors when offered food is not taken was calculated as the rate of looking at (or for) the partner during focal events *minus* looking at (or for) the partner during matched control events

This criterion was assessed in the dyadic game in the Kalium group (Fig. 2, videos from Burkart et al. 2007). First, we selected as focal events all those test trials when donors had pulled the tray so that the food was available for the recipient but the recipient did not take the food until the end of the trial (maximum trial duration 30 s, Fig. 4). We coded the behavior of the donor after the pull until the end of the trial (i.e. during the focal period). Next, for each such focal event, we chose a control event, which was defined as the temporally closest trial of the same dyad in which the individual in the donor role did not pull the tray. In these control events, we coded the behavior of the individual in the donor role (same individual as in the focal event) during the same time window as in the focal events. In particular, we coded whether the focal individuals looked *at* the potential recipient, or *for* the potential recipient, by moving closer to the window and watch the other side, when the latter was out of the visual field of the donor. During the focal period, the situation was thus identical: both animals were sitting in their compartment, and no one was eating. The only difference was that during the focal test periods, this was preceded by the focal individual offering the food, which would still have been available for the reluctant recipient. Unfortunately, reliabilities could not be implemented here because the videos were no longer accessible. However, looking directions of the common marmosets can easily be identified based on the hair tufts that are situated around the ears, and we usually achieve very high reliabilities when doing so (e.g. Burkart et al. 2012: inter-rater agreements between 96%–99.5%, correlations > 0.96 for gaze durations; Kupferberg et al. 2013: inter-rater agreements between 96% and 98% for gaze durations, and Miss and Burkart 2018: Cohen’s Kappa for gaze frequencies are between 0.72 and 0.86).

First, we used LMMs to assess whether the subjects were more likely to look at and to look for the partner during the focal event compared to the matched control event, and used the proportion of the event during which the donor monitored its partner as dependent variable, condition as fixed factor, and identity of the donor and of the recipient, dyad, and event nested in dyad as random factors. Second, we quantified the strength of the reaction of the donor to the unexpected outcome of his behavior (Fig. 4), i.e. that the recipient would not take the provisioned food, by calculating the difference of the rates of looking at/for the partner in focal events minus control events. This strength of reaction to the unexpected outcome was then compared to an individual’s prosociality as assessed in the dyadic game (data from Burkart et al. 2007), in a GLMM with the strength of the reaction to the unexpected outcome as dependent variable, prosociality as fixed factor and the same random factors as above.

## Results

### Test 1: audience effects in the group service paradigm

When testing the marmosets with the group service task with and without an audience, the behavior of the marmosets was consistent with the presence of an audience effect, i.e. they pulled the tray more often in the audience-present condition. In the no-audience condition, pulling occurred in 16% of all trials and in the audience condition in 56% of all trials (*χ*^2^(1) = 8.68,* p* = 0.003). 86% of all pulls in the audience-present condition were coordinated and resulted in a food transfer. Thus, in the audience-present condition, food provisioning occurred in 48% of all trials. These results are thus consistent with the presence of an audience effect in the group service test. Note that this provisioning rate is similar to the last regular test of phase IV in this group of marmosets (data from Burkart et al. 2014), where food provisioning occurred in 55.7% of all trials: audience condition (test 1) vs. last regular test of phase IV (from Burkart et al. 2014): *χ*^2^(1) = 0.32,* p* = 0.57).

### Test 2: persistence in the group service paradigm

**Fig. 5:**
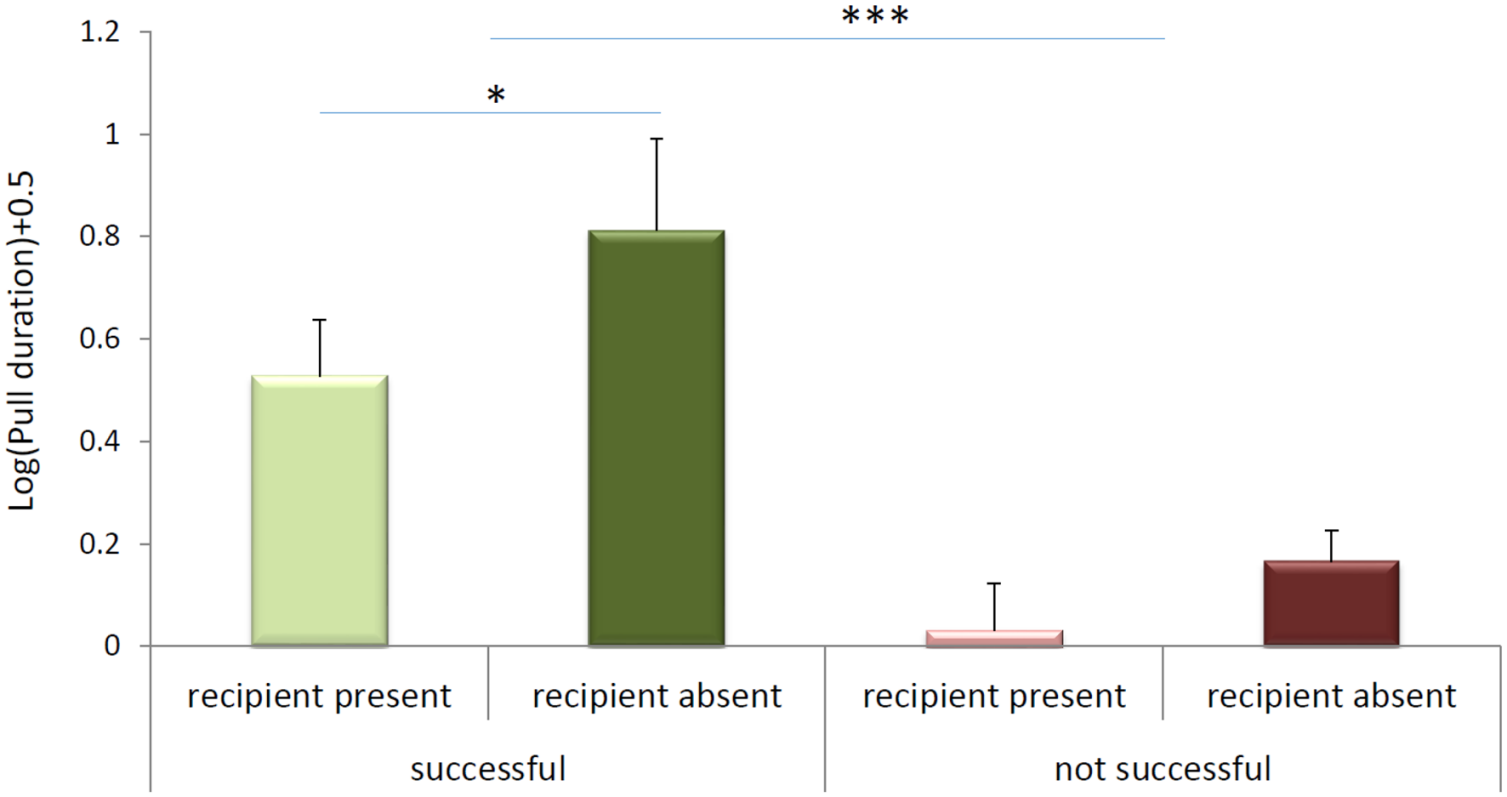
Pull durations. Pulls can be successful (i.e. lead to a transfer), or non-successful. Moreover, when the pull starts, a potential recipient can already be present in the recipient position or not. Successful pulls are significantly longer than non-successful ones, and they are particularly long when the recipient first has to approach the recipient position

In the group service experiment, where the tray had to be held in place until a group member retrieved, pulls that led to a transfer were significantly longer than pulls that did not, regardless of whether a potential recipient was already present close to the recipient position or not (LMM with individual as the random factor: *F*_(3,12,2)_ = 10.1, *p* < 0.001). The longest pulls were observed when successful transfers occurred but the recipients first had to approach the apparatus (Fig. 5, LSD post-hoc tests), consistent with the idea that donors pulled the apparatus and waited for the recipient to come and get the food.

**Fig. 6:**
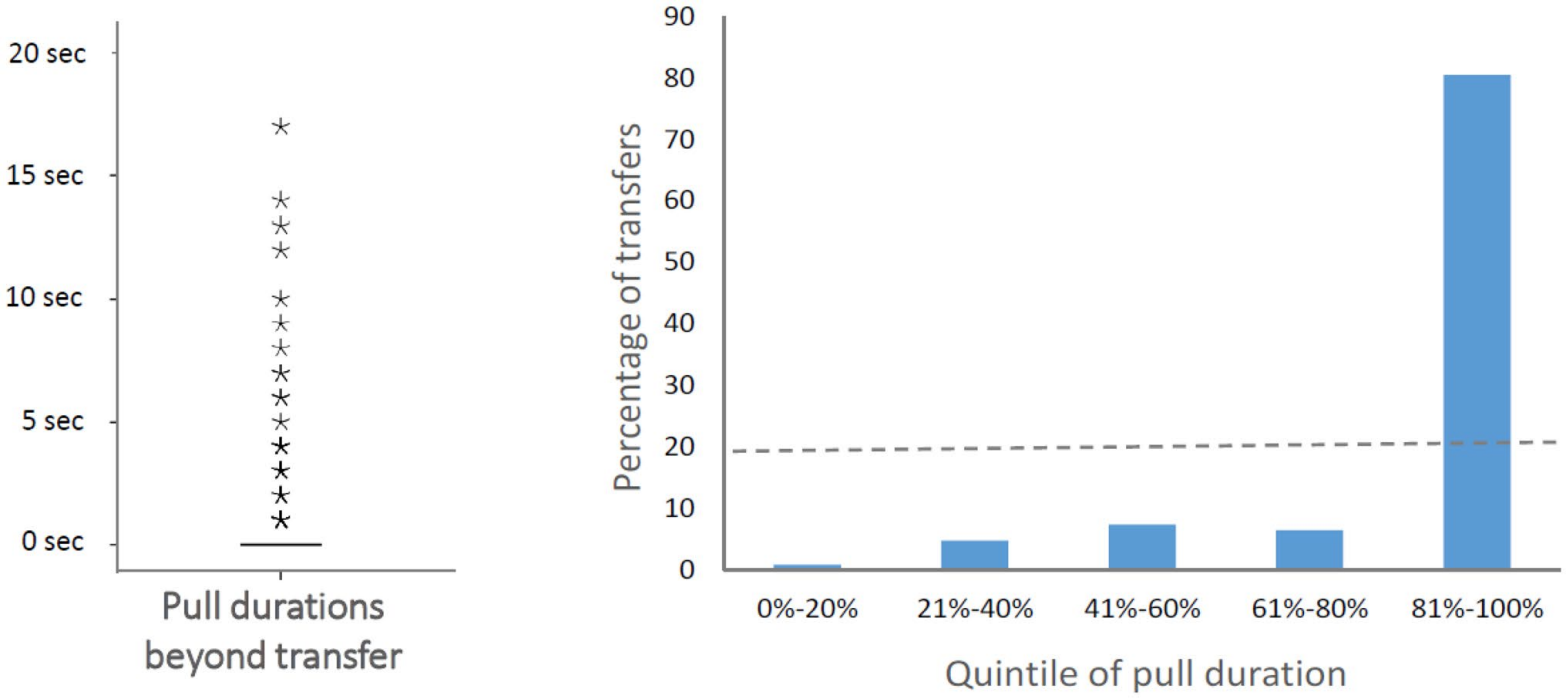
Pull durations relative to transfers. *Left*: boxplot of all durations between transfers and release of the handle (median and quartiles = 0, visible are only outliers). *Right:* distribution of transfers across the entire duration of a pull. The majority of all transfers occurred in the last 20% of the duration of the corresponding pull. The dotted line represents the expected distribution if the transfers were randomly distributed over the duration of the pulls

Obviously, during longer pulls, successful transfers would also be more likely by chance. We, therefore, performed a second set of analyses aimed at identifying whether the potential donors in the group service paradigm continued to pull (i.e. kept the board near the mesh so recipients could take the food) exactly until the recipient had retrieved the food, or whether the pull duration was independent of the timing of the transfer. The vast majority of all pulls (i.e. 78.8% of 231 food transfers) stopped in the very same second when the food had been retrieved, and 87% stopped in the same or the next second; the donors thus usually didn’t continue to pull beyond the moment of the transfer (Fig. 6, left). The distribution of transfers relative to the duration of a pull is shown in Fig. 6 (right) and is significantly different from a random distribution when calculated across all subjects (*χ*^2^_(4)_ = 531.6,* p* < 0.001), and also when calculated per individual for those individuals who transferred food often enough to allow for statistical analysis (*χ*^2^_(4)_ between 422.8 and 18.66, *p* < 0.001 in all cases). These results are consistent with the hypothesis that the marmosets pulled the tray in a goal-directed way and engaged in response waiting by holding on to the board exactly until the putative goal of transferring the food to group members was reached.

### Test 3: are marmosets surprised if the food they provide to a partner is not taken? Audience checking

Finally, we analyzed whether the marmosets in the dyadic game showed a distinct reaction when their behavior of pulling the tray within reach did not result in the putative goal of providing food to others. To do so, we analyzed the behavior of the donors after they pulled the tray, but for some reason, the recipient would not take the food. Overall, the marmosets tended to look at or for the partner more in the focal events compared to the matched control events (*F*_1,55.7_ = 3.65, *p* = 0.061). However, not all marmosets are equally prosocial and some, in particular female helpers, didn’t show significantly more pulling for their partners compared to the non-social control condition. We, therefore, expected that prosocial individuals (i.e. those with a higher difference score indicative of the extent of proactive prosociality) should be the ones showing the strongest reaction to partners not taking the provisioned food. We thus correlated the strength of the reaction to the unexpected outcome (i.e. that the partner would not retrieve the provisioned food), to variation in the prosociality difference score. These results show that the more prosocial a donor behaved in the prosocial game toward a specific recipient (Burkart et al. 2007), the stronger the donor reacted by looking at and looking for the recipient when this recipient did not retrieve the food (*F*_1,15.44_ = 7.506, *p* = 0.015).

**Fig. 7:**
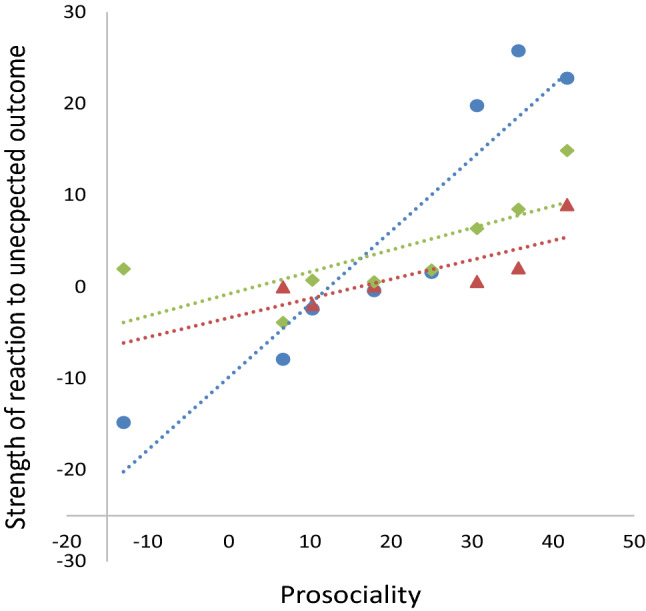
Correlation between the strength of the reaction of a donor when a recipient would not retrieve the provisioned food and its proactive prosociality as assessed in the dyadic game (quantified as difference score, i.e. pulling the tray when a partner is present minus pulling the tray in non-social control conditions). Blue circles: looking at the recipient, red triangles: looking for the recipient when it had moved out of the donor’s visual field, green diamonds: combination of both measures

Importantly, this pattern was not driven by a general preference of the donor to look at this specific recipient, because a relationship between looking at and for the partner was only present during the focal events (*F*_1,15.12_ = 7.19, *p* = 0.017) but not during matched control events (*F*_1,40_ = 0.036, *p* = 0.85). Figure 7 illustrates this relationship between the strength of the reaction to the unexpected outcome per individual, separately for looking at the partner (Rho = 0.976, *n* = 8, *p* < 0.001), looking for the partner (Rho = 0.886, *n* = 6, *p* = 0.019), and the combined measure (Rho = 0.738, *n* = 8, *p* = 0.037).

## Discussion

The aim of this paper was to examine to what extent prosocial behaviors in marmoset monkeys fulfil key criteria of intentionality that have been developed in the context of primate communication (Townsend et al. 2017, summarized in Table 1). The results reported here provide evidence for the criteria in Table 1 (***bold italics***) that couldn’t be assessed previously based on the available literature. Existing and new tests thus provide strongly convergent evidence for markers of intentionality in marmoset prosocial behavior in both naturalistic and experimental contexts.

First, marmoset prosociality shows some degree of flexibility, both in the wild and in captivity. The best proxy for proactive prosociality under naturalistic conditions is proactive food sharing because it is unambiguously initiated by the food possessor and can be performed ad libitum by all group members. Patterns of proactive food sharing suggest that this is more than an automated reaction to food during periods when immatures are present in the group, because adult food providers take into account how difficult it is for immatures to obtain the food, independent of their age (Guerreiro Martins and Burkart 2013; Humle and Snowdon 2008; Moura et al. 2010; Price and Feistner 1993; Rapaport 2011). Furthermore, marmosets can use different means to engage in provisioning, including novel experimental apparatuses in dyadic provisioning games and the group service task that require a pulling response, which is not part of the naturalistic motor patterns involved in provisioning. This means-end dissociation is one of the key criteria for intentionality in particular in developmental psychology (Piaget 1954; Bard 1992; Cartmill and Byrne 2010; Leavens et al. 2005; Liebal et al. 2013; Tomasello et al. 1994), because it indicates that actors select the most appropriate actions from among a range of possible actions to reach the actual goal (here: provisioning the recipient).

Second, audience effects have been shown in naturalistic contexts when emitting food-offering calls. Calls were more frequently emitted when group members were out of sight, and, therefore, had to be informed about the presence of a valuable food source (Vitale et al. 2003). Furthermore, marmosets proactively share more food with immatures when they alone are responsible for them (Brügger et al. 2018). Audience effects were also present in both experimental contexts. In prosocial games, the presence of audience effects on prosocial behavior is a necessary condition to demonstrate proactive prosociality in this experimental context (Cronin 2012; Marshall-Pescini et al. 2016). Likewise, we demonstrated audience effects with test 1 in the group service context (***this study***), where the marmosets were more likely to pull the board when potential recipients were present in the recipient compartment. This result is not surprising, since in the group service experiments, the quantification of proactive prosociality had been implemented with a set of control conditions that also addressed the social use of the behavior (Burkart and van Schaik 2013 and Burkart et al. 2014). However, the audience-present vs. absent test as implemented here has an additional advantage. This design also controls the possibility that experimentally assessed prosociality effects are an artifact of social facilitation (Jensen et al. 2006; Silk et al. 2005). Social facilitation (Zajonc 1965) increases the probability that an individual shows more behavior due to the mere presence of conspecifics, and thus constitutes an alternative explanation inherent to dyadic games where social conditions are compared with non-social control conditions. Note that in the audience-absent condition implemented here, the density of animals in the compartment around the handle is twice as high as in the audience-present condition (where half of the group was in the second compartment, close to the recipient position). Thus, social facilitation effects should have been stronger in the audience-absent condition, but individuals nevertheless pulled the tray less often compared to the audience-present condition. This outcome is, therefore, in line with the presence of an audience effect, indicative of the willingness to provision food, which in turn indicates voluntary control over the pulling actions.

Third, the behavior of marmosets during prosociality tasks is consistent with criteria for goal-directedness. First they pursue a social goal and persist until they have reached that goal (Test 2, ***this study***). In the group service context, the marmosets pulled and held the tray exactly until the food had been retrieved from the board by a group member. The stopping rule that can be inferred from this pattern is to pull until the food has been taken. Second, (Test 3, ***this study)*** prosocial individuals in the dyadic games checked back at the audience when it did not behave as expected, i.e. retrieve the provisioned food. In the dyadic games, results show that the more prosocial donors are (i.e. the more food they provide to the partner), the more they check back at the recipient if the latter does not retrieve food that the donor has pulled within her reach. The donor reacts to the reluctance of the recipient to take the food by looking at her, or by looking for her if she has moved out of the immediate visual field, by walking to a position from where the recipient can be seen. This back-checking is particularly strong in more prosocial individuals. All of this suggest surprise at the unexpected outcome, and thus that the donor intends to provide food to the recipient.

Marmoset prosocial behavior thus satisfies multiple markers of intentionality related to the criteria of flexibility (behavioral adjustment, means-ends dissociation), social use (audience effects), and goal-directedness (response waiting, audience checking), which have been developed to identify intentionality in nonhuman primate communication. We thus find convergent evidence from different contexts, which makes an intentional interpretation of the behavior increasingly likely. Nevertheless, it is important to stress that this approach is limited as it only allows us to infer that the behavior in question is consistent with an intentional description. It is thus vulnerable to more mechanistic alternative explanations, in particular because this approach does not allow us to falsify the hypothesis that a specific behavior is under intentional control. For instance, if a communicative act or behavior does not satisfy one of the several criteria that have been proposed, it is not equivalent to evidence that this behavior is not under intentional control.

What is critically lacking, therefore, is a direct behavioral test that can provide both positive and negative evidence for whether a behavior is produced intentionally. Providing such direct evidence for intentionality in action production is extremely challenging. However, another direction of research has investigated whether nonhuman animals perceive, rather than produce, behavior intentionally (Call et al. 2004; Phillips et al. 2009; Rochat et al. 2008; Wood et al. 2007). This work has in particular been inspired by developmental psychology where preverbal children’s action understanding has been investigated based on habituation–dishabituation paradigms (Baillargeon et al. 2014). This approach has also been applied to marmoset monkeys. Based on the paradigm developed by Amanda Woodward (Woodward 1998, 2009), it could be shown that marmosets do indeed perceive the behavior of conspecifics as goal-directed, rather than responding to surface properties of the behavior (i.e. physical properties such as movement trajectory). Furthermore, the marmosets only learned socially from entities that they had previously perceived as behaving in a goal-directed way (i.e. a conspecific, and to a lesser extent a monkey-like robot, but not from a black box: Burkart et al. 2012; Kupferberg et al. 2013).

Taken together, the available evidence shows that marmoset prosociality fulfils several criteria of intentional action and that furthermore, they also perceive another’s actions as intentional (i.e. goal-directed) and that this perception is guiding further behavioral decisions. This adds to the increasing number of studies providing evidence for intentionality in a broad number of species (Townsend et al. 2017), including fish (Vail et al. 2013) and birds (Ben Mocha and Pika 2019). Moreover, the intentionality may have extended to other contexts beyond food calls, food sharing and provisioning. Different kinds of prosocial behaviors tend to be correlated in cooperative breeders (e.g. Madden and Clutton-Brock 2010) and all appear to be regulated by oxytocin, including in common marmosets (Finkenwirth et al. 2016).

Overall, then, the most parsimonious conclusion to date is that marmoset prosociality is under some form of intentional control and is, therefore, not fundamentally different from human prosociality with regard to this aspect. This conclusion does not imply that every instance of a prosocial behavior in marmosets is under constant intentional control (which is also not the case in humans: Hawley 2014), or that there are no differences between marmoset and human prosociality. In particular, the far more sophisticated cognitive abilities of humans compared to marmosets most likely allow for more top-down control and a more complex integration of specific social and environmental conditions, as well as additional behavioral goals. For instance, well developed cognitive empathy and Theory of Mind abilities help in identifying the exact need of an individual, which obviously goes beyond the need for food, as well as the behavioral means that are most likely to fulfil these needs. These cognitive abilities are likely to constrain the range of helping behaviors that an individual can engage in, not only in marmosets, but also in chimpanzees (Warneken 2013) and young children (Vaish and Warneken 2012).
